# Mechanisms of Neurotoxicity Associated with Exposure to the Herbicide Atrazine

**DOI:** 10.3390/toxics9090207

**Published:** 2021-08-31

**Authors:** Sydney C. Stradtman, Jennifer L. Freeman

**Affiliations:** School of Health Sciences, Purdue University, 550 Stadium Mall Drive, West Lafayette, IN 47907, USA; sstradtm@purdue.edu

**Keywords:** atrazine, brain, central nervous system, crosstalk, dopamine, endocrine disruption, HPG axis, HPA axis, neurotransmitters, neuroendocrine

## Abstract

Atrazine is an herbicide commonly used on crops to prevent broadleaf weeds. Atrazine is an endocrine-disrupting chemical mainly targeting the neuroendocrine system and associated axes, especially as a reproductive toxicant through attenuation of the luteinizing hormone (LH). Current regulatory levels for chronic exposure are based on no observed adverse effect levels (NOAELs) of these LH alterations in rodent studies. Atrazine has also been studied for its effects on the central nervous system and neurotransmission. The European Union (EU) recognized the health risks of atrazine exposure as a public health concern with no way to contain contamination of drinking water. As such, the EU banned atrazine use in 2003. The United States recently reapproved atrazine’s use in the fall of 2020. Research has shown that there is a wide array of adverse health effects that are seen across multiple models, exposure times, and exposure periods leading to dysfunction in many different systems in the body with most pointing to a neuroendocrine target of toxicity. There is evidence of crosstalk between systems that can be affected by atrazine exposure, causing widespread dysfunction and leading to changes in behavior even with no direct link to the hypothalamus. The hypothetical mechanism of toxicity of atrazine endocrine disruption and neurotoxicity can therefore be described as a web of pathways that are influenced through changes occurring in each and their multiple feedback loops with further research needed to refine NOAELs for neurotoxic outcomes.

## 1. Introduction

The herbicide atrazine (6-chloro-*N*-ethyl-*N*-(1-methylethyl)-1,3,5-triazine-2,4-diamine) is one of a family of chemicals known as triazines and is in the top two herbicides used in the United States [1,2]. Triazines are historical for being endocrine-disrupting chemicals and research has supported this hypothesis in regard to atrazine specifically. Atrazine is used on crops in the United States and other global regions for the control of both broadleaf and grassy weeds [1]. Any population living in close proximity to where atrazine is applied, such as in the United States Midwest, is particularly vulnerable to atrazine exposure due to the large land masses used to farm crops, such as corn, that are treated with the chemical [3]. The most common route of environmental exposure to this toxicant is through ingestion of contaminated drinking water, while inhalation and dermal absorption can occur in occupational settings during the production of atrazine as well as the application to crops [1,3,4]. The European Union (EU) recognized the potential harm that atrazine has on public health regarding its endocrine-disrupting nature and involvement in adverse reproductive and development outcomes. Therefore, in 2003, the EU reevaluated a potential ban on the use of atrazine. The herbicide was not approved for use to prevent drinking water contamination, because of the difficulty in containing the chemical when in use, often exceeding the concentration limit set [5]. Recently, in the fall of 2020, the United States Environmental Protection Agency (US EPA) reapproved atrazine for use as well as eliminated some of the requirements of monitoring atrazine in drinking water by doing away with the Atrazine Monitoring Program (AMP). The agency states that the concentrations that are regularly measured are routinely low with the highest recorded atrazine concentration in drinking water being much lower than the limit set for triazines. However, the Atrazine Ecological Exposure Monitoring Program (AEEMP) will still be continued because concentrations in the environment pose ecological risk [6]. The US EPA maximum contaminant level (MCL) for atrazine in drinking water is 3 parts per billion (ppb, µg/L), but in contrast to the above statements, others report it is likely that during peak crop seasons, when use is most abundant, this regulatory limit can be exceeded both in ground and surface drinking water sources [2,3]. In addition, the World Health Organization’s (WHO) current regulatory limit for atrazine in drinking water is much higher, set at 100 ppb.

Atrazine has been extensively studied for years in regard to its adverse developmental and reproductive outcomes in many different models, in vivo and in vitro, with the current no observed adverse effect level (NOAEL) at 10 mg/kg/day and the LOAEL at 70 mg/kg/day for acute dietary exposure and the NOAEL at 1.8 mg/kg/day and the LOAEL at 3.65 mg/kg/day for chronic dietary exposure based on neuroendocrine impacts [7,8,9,10,11]. Many epidemiological studies have also found correlations and associations between atrazine exposure and outcomes such as preterm delivery and small-for-gestational-age, birth defects, and other adverse birth outcomes. The region of focus for many of these studies typically tends to be populations residing in the US Midwest where drinking water contamination is high and proximity to crop fields where atrazine is used is more prevalent [1,3,12,13,14,15,16]. Other epidemiological studies have shown an association between atrazine exposure at concentrations below the MCL set by the US EPA with the alteration of hormones involved in the menstrual cycle as well as reduced semen quality in areas proximal to pesticide usage [17,18]. Recent research is beginning to look at other areas of the brain that may be affected and how the dysregulation of different systems caused by atrazine are interconnected with the reproductive outcomes seen.

Broadly, atrazine exposure is known to affect the neuroendocrine system and associated endocrine axes, specifically the hypothalamus-pituitary-gonadal (HPG) axis. Studies have shown adverse effects on pubertal development of rats, mammary gland development in prenatally exposed offspring, changes in luteinizing hormone (LH) surge and gonadotropin-releasing hormone (GnRH) release in female rats, and delayed vaginal opening associated with delayed puberty [8,19,20,21,22,23]. Other research on male organismal models have shown that the male offspring of dams treated with atrazine during pregnancy had drastically reduced levels of testosterone, while other studies showed that maternal toxicity did not affect the male reproductive development. Zebrafish models have shown a multitude of effects in both females and males including impairment in the release of reproductive hormones, head and body morphology, decreases in spawning, and dysfunction in gene expression associated with behavior and development [24,25,26]. Although there is much known about the effects of atrazine as a reproductive toxicant and endocrine-disrupting chemical in regard to the hypothalamus, specifically the HPG axis, the exact mechanism (or mechanisms) of toxicity have yet to be elucidated. Studies are beginning to explore the hypothalamic-pituitary-adrenal (HPA) axis as well as the central nervous system’s role with neurotransmitters and how these adverse outcomes are connected through crosstalk between systems in the brain [27,28,29,30,31,32]. The research on atrazine as a neurotoxicant is limited at this time and has been waived for consideration in human health risks assessments by the US EPA. However, the studies that are available regarding adverse effects on neurotransmission suggest that atrazine neurotoxicity is worth exploring, because of the potential for abnormal neurotransmitter release and dysfunction to cause neuropsychiatric and neurodegenerative diseases [33].

**Figure 1:**
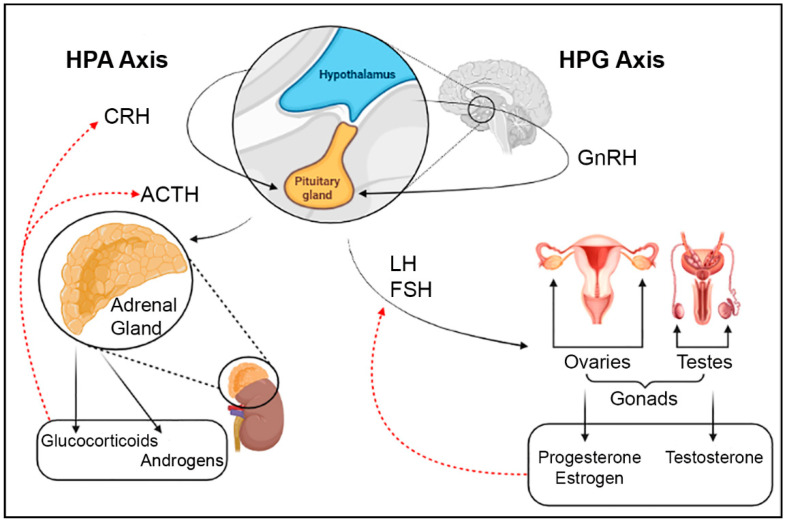
Outline of the pathways of HPG and HPA axes originating at the hypothalamus. Red dotted arrows represent the feedback loops that the specific components of the pathways regulate. Image created in BioRender. [ACTH: adrenocorticotropic hormone, CRH: corticotropin-releasing hormone, FSH: follicle stimulating hormone, GnRH: gonadotropin-releasing hormone, LH: luteinizing hormone].

The hypothalamus is an important region of the brain that plays a major role in the neuroendocrine system and is of particular interest in regard to atrazine exposure [7,8,9,27,28,30,34]. The hypothalamus is responsible for the production and release of seven neurohormones, including GnRH, corticotropin-releasing hormone (CRH), and dopamine [35]. These neurohormones are produced by neuroendocrine neurons that are prevalent in two regions of the hypothalamus, but more specifically, form the paraventricular hypothalamic nucleus (PVH), periventricular nucleus (PV), supraoptic nucleus (SO), and the arcuate nucleus (ARH) [34]. There are three axes in which the hypothalamus and the pituitary are involved that allow for the synchronicity of neuronal systems, the central nervous system, and the endocrine system. The literature shows that the HPG and HPA axes are most commonly studied in relationship to their adverse outcomes following atrazine exposure [7,29]. HPG axis communication starts at the hypothalamus, where GnRH is released to activate the anterior pituitary to secrete LH and follicle stimulating hormone (FSH) [7] (Figure 1). These hormones further trigger the production and release of sex steroids that negatively feedback to regulate this cycle [36]. While the HPG axis is typically involved in reproduction, the HPA axis is responsible for the regulation of stress response [29]. The HPA also begins at the hypothalamus with the release of CRH, which then activates the anterior pituitary to secrete adrenocorticotropic hormone (ACTH) to travel through the blood to the adrenal cortex where the synthesis of glucocorticoids occurs [29] (Figure 1). There is evidence of crosstalk between these two axes in regard to how the gonadal hormones help regulate responses to stressors, including both chronic and traumatic stressors, and dysregulation of either of these axes can affect the other, potentially leading to disorders such as anxiety, depression, and post-traumatic stress disorder [29].

The scope of this review is to examine the mechanisms of atrazine toxicity involving both the neuroendocrine system and the associated hormones, as well as the central nervous system in regard to multiple major neurotransmitters. The hypothalamic axes of the neuroendocrine system that will be discussed in this review are limited to the HPG and HPA axes, but it should be noted that ongoing research is also evaluating impacts along the hypothalamus-pituitary-thyroid axis, although this body of research at this time is more limited.

## 2. Role of Major Neuroendocrine Hormones Regulated by the Hypothalamic-Pituitary Axes

The harmonious interaction of the hypothalamus in the CNS as well as multiple glands in the endocrine system gives rise to the study of the complex system known as the neuroendocrine system. The hormones involved in the neuroendocrine system are specifically associated with different hypothalamic axes [37]. The functions of these hormones are discussed in detail to provide an understanding of their importance when referenced in regard to atrazine exposure.

### 2.1. Hypothalamic-Pituitary-Gonadal Axis

#### 2.1.1. Gonadotropin-Releasing Hormone (GnRH)

GnRH is a critical neurohormone released by the hypothalamus in the regulation of reproductive function. Release of GnRH from the hypothalamus into the portal circulation allows it to reach the anterior pituitary. GnRH is released in a pulsatile mode, where there are specific events in which it is released, as well as a surge mode, where there is a constant concentration of GnRH prior to ovulation in females [38,39]. Once GnRH reaches the anterior pituitary, the secretion of gonadotropins LH and FSH are triggered. In the event that the normal pulsatile release of GnRH is disrupted and constant surges occur when not appropriate, the GnRH receptors can become desensitized and inactive in the anterior pituitary, causing a direct impact on the secretion of LH and FSH [38].

#### 2.1.2. Luteinizing Hormone (LH)

LH, as mentioned, is released by the anterior pituitary following stimulation by GnRH [40]. Typically, low pulse frequencies of GnRH are associated with the synthesis and release of LH [41]. LH is an important neurohormone during development because of its contribution to the evolution of immature germ cells into mature cells for both males and females. There are other roles of LH that do differ between sexes. In males, LH prompts the production of testosterone in Leydig cells, while in females LH is responsible for prompting the synthesis of steroid hormones such as estrogen and progesterone. Testosterone, estrogen, and progesterone then also negatively feedback to control the concentration of LH that is secreted creating a loop. These pathways are important in a mature organism and also in the developing organism. Stability of these pathways during developmental stages is key, because LH is important in the reproductive development of an organism [40].

#### 2.1.3. Follicle-Stimulating Hormone (FSH)

FSH, along with LH, is released after stimulation of the anterior pituitary by GnRH from the hypothalamus. As previously mentioned, high pulse frequencies of GnRH from the hypothalamus are associated with the synthesis and secretion of LH and in contrast, low pulse frequencies are typically associated with the synthesis and secretion of FSH. FSH in developing males is responsible for the increase in production of Sertoli cells, which amount to a large portion of the testes. Proliferation of the Sertoli cells is important, because they produce a hormone that prevents female genitalia from forming inside the organism. In female development, FSH aids in the development of the ovarian follicles. In mature organisms, FSH plays a slightly different role. In the mature female, FSH triggers aromatase synthesis in the ovaries, which is useful in the modification of androgens to estradiol. In mature males, FSH regulates the production and function of sperm in conjunction with testosterone [41].

### 2.2. Hypothalamic-Pituitary-Adrenal Axis

#### 2.2.1. Corticotropin-Releasing Hormone (CRH)

CRH is the main neurohormone that regulates the HPA axis through its response to stressors. When stress is experienced, the hypothalamus is triggered to synthesize and secrete CRH, which travels to the anterior pituitary to signal for the synthesis and release of ACTH. CRH also activates the expression of proopiomelanocortin (POMC). Aside from triggering the release of ACTH, the CRH plays a role in the immune system as well, being activated by not only stress, but also cytokines [42]. CRH provides linkage of the HPA axis across systems.

#### 2.2.2. Adrenocorticotropic Hormone (ACTH)

ACTH is controlled by the hypothalamus through the secretion of CRH after exposure to stressors, as previously mentioned. ACTH’s assigned destination organ is the adrenal cortex where it triggers the secretion of glucocorticoids and androgens. The glucocorticoids then help regulate both ACTH and CRH through a negative feedback loop to mediate the response to stress [43].

## 3. Effects of Atrazine on Major Neuroendocrine Hormones

Atrazine is known to be an endocrine-disrupting chemical having effects on the signaling pathways of the neuroendocrine system previously discussed. The system of importance when reviewing adverse health outcomes is typically the reproductive system since many of the neuroendocrine hormones affected play a role in different aspects of reproduction. There can be difficulty in defining mechanisms of atrazine as an endocrine-disrupting chemical, because there are many factors that play a role in the adverse outcomes that are observed including when the exposure occurs, the length of the exposure, as well as the dose or concentration of the exposure. Many different models have been used to explore the effects of atrazine exposure including various cellular systems, rodents, and zebrafish. There have also been multiple different time points studied, because atrazine has effects on the development of organisms as well as effects on mature adult organisms [7].

### 3.1. HPG Axis

The hypothalamus is of particular importance when studying atrazine due to the neuroendocrine outcomes that are continuously proven in the literature [7,8,9,27,28,30] (Table 1). The hypothalamus is abundant in GnRH neurons that produce and release the neurohormone GnRH [9,35]. A study done on the exposure of atrazine at 200 mg/kg in female rats revealed that GnRH release is inhibited following exposure to atrazine, directly affecting the synthesis of LH and FSH [7,8]. Downstream, the dysregulation of these gonadotropins can impact the secretion of sex hormones [44]. This study also suggested that the secretory aspect of GnRH was affected independently of protein expression and mRNA levels, so there is likely an upstream target of the GnRH neurons that atrazine is targeting [8]. An additional study with female Wistar rats confirmed the adverse effects that were likely to be seen on the LH surge, because of the known outcomes of atrazine on GnRH. An atrazine exposure of 4 days at 50, 100, or 200 mg/kg caused a decrease in the LH surge, but after withdrawal of exposure, levels of GnRH and LH were stabilized to normal levels [9]. Although levels of these important neuroendocrine hormones have the potential to regulate themselves after an exposure has commenced, small windows of exposure like the one tested in the mentioned study, can have the potential to cause pathology and dysfunction if occurring at a critical time during development. Other studies looking at ovariectomized female rats found that the LH surge was decreased after a series of exposures to atrazine. A single exposure at the lower doses of 10, 30, or 100 mg/kg showed an increase in the LH surge as well as an increase in *Kiss1* expression in the anteroventral periventricular nucleus (AVPV) following a 1 h exposure, suggesting that atrazine also plays a role in changing the surge [45]. The *Kiss1* gene is responsible for regulating GnRH and in a similar study in the same strain of rats, *Kiss1* was inhibited by a series of atrazine exposures at 100 mg/kg in the AVPV [46]. A decrease in LH levels following atrazine exposure was also witnessed in male rats exposed to atrazine between 1 and 200 mg/kg and was suggested to be the cause of the decrease in testosterone in the same study [47]. Seeing changes in LH across both male and female rat models supports the hypothesis that the hypothalamus is the target of atrazine causing many downstream effects. Furthermore, studies have also shown decreases in FSH in male rats and female rats exposed to 100, 200, or 400 mg/kg atrazine. This finding is expected based on the relationship of FSH to GnRH with studies in zebrafish confirming a decrease in GnRH after an embryonic exposure to 0.3, 3, or 30 ppb (µg/L) atrazine via immersion [26,48]. Overall, the cumulative body of research in this area confirms a wide range of atrazine exposure doses over short and longer term durations attenuates LH surge and disrupts estrous cyclicity. Based on these observations the US EPA anchored the dietary chronic exposure to the attenuation of the LH surge with a NOAEL at 1.8 mg/kg/day and the LOAEL at 3.65 mg/kg/day. These studies focused on the reproductive outcomes are reviewed in more detail in [7].

### 3.2. HPA Axis

Although the focus of atrazine toxicity is typically on the HPG axis, a few studies have now also shown that the HPA axis function is affected by atrazine exposure as well. Brain transcriptomic data of adult male zebrafish exposed to 0.3, 3, or 30 ppb atrazine only during embryogenesis revealed that the HPA axis may also be affected [26]. In addition, a study in female rats discovered that atrazine exposure of 75 mg/kg caused an increase in the pituitary hormone ACTH [28]. A more recent study explored the CRH receptors as toxicity targets and found that exposure to atrazine or the chlorometabolites, desethylatrazine (DEA) and deisopropylatrazine (DIA), at 100 mg/kg caused an increased release of CRH into the vasculature, which was responsible for the increase in ACTH [27]. The HPA axis is responsible for responding to stressors and triggers the synthesis and release of these hormones [30]. Overall, there is limited research available on the HPA axis and atrazine in general, but one study did provide evidence that exposure in adult male rats showed an increase in ACTH at a LOEL of 12.5 mg/kg atrazine and at 135 mg/kg diamino-s-chlorotriazine (DACT), a major metabolite of atrazine [49]. Another study found proteomic changes of adrenal proteins and testis proteins that correlate to testosterone found in serum in both intact and castrated male rats after exposure to atrazine, further supporting the HPA axis as a target of atrazine toxicity [50], but additional research is needed to further the understanding of atrazine’s potential impacts along this axis.

## 4. Role of Major Neurotransmitters in the Hypothalamus

Neurotransmitters are important signaling molecules of the CNS that are produced by specific neurons located in particular parts of the brain. In regard to brain function, neurotransmitters are an essential aspect of the system, helping regulate basic bodily functions, maintaining homeostasis, response to stress, motor functions, emotions, and more. Each neurotransmitter is responsible for relaying information between neurons so the brain and other parts of the body can respond accordingly to different events the body is experiencing. The CNS is also an adaptive system having the capability of forming memory to be able to respond more adequately to similar scenarios in the future [51].

The hypothalamus is a unique part of the brain playing a role in the endocrine system as previously discussed, but also in the CNS. Some neurons are located within the hypothalamus, while other neurons have projections into this brain region. These neurons are involved in the production and release of key neurotransmitters that signal the hypothalamus to carry out its functions. Temperature regulation, autonomic nervous system regulation, as well as appetite control are some of the bodily functions that the hypothalamus regulates that are non-endocrine functions. The disruption of these key neurotransmitters in signaling and sending information to the hypothalamus not only disrupts its role in neuroendocrine functions, but also in other homeostatic functions and has the potential to lead to different pathologies [52].

### 4.1. Dopamine

**Figure 2:**
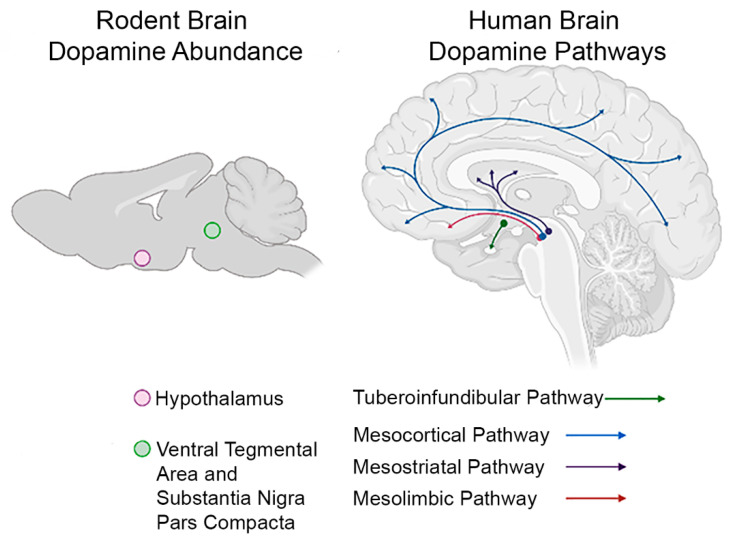
Dopamine distribution and pathways in the rodent and human brain. Dopamine neurons are located within the hypothalamus and the ventral tegmental area (purple circle) and substantia nigra pars compacta (green circle) in the rodent brain. In the human brain, dopaminergic neurons are located within the tuberoinfundibular (green), mesocortical (blue), mesostriatal (purple), and mesolimbic (red) pathways. Location of dopaminergic neurons within these multiple brain regions provides support that an environmental chemical contaminant (e.g., atrazine) targeting the dopaminergic system may impact multiple brain regions and thus multiple functions. Image created in BioRender.

Dopamine is an important neurotransmitter that balances certain processes of the CNS including learning, memory, motor activity, and motivational behaviors in the adult brain [53]. The pathways needed for the mentioned behaviors and movement are impacted by dopamine during development as well. Dysregulation in dopamine and signaling can affect neurocircuitry by altering certain neurons [54]. The substantia nigra, ventral tegmental area (VTA), and the hypothalamus are the three main areas of the brain where dopamine is produced and released to the striatum, nucleus accumbens, and the pituitary, respectively [55,56] (Figure 2). This release occurs through the dopaminergic pathways known as the nigrostriatal pathway, mesolimbic pathway, mesocortical pathway, and the tuberoinfundibular pathway [35,57]. Dopamine synthesized in the hypothalamus by the tuberoinfundibular (TIDA) neurons is of particular importance in the neuroendocrine system. Dopamine is typically considered a neurotransmitter, but in regard to the tuberoinfundibular pathway, originating in the hypothalamus, dopamine is considered a neurohormone, because of its release into the pituitary portal system to inhibit the secretion of a pituitary hormone, prolactin [35]. The TIDA neurons controlling hypothalamic dopamine synthesis and release are found in a very specific region of the hypothalamus known as the arcuate nucleus, whose main function is to regulate homeostasis and pathways that control food intake, energy usage, and body weight [58]. The importance of knowing the role of the arcuate nucleus in relationship to both dopamine and atrazine exposure will be further discussed below. In addition, dopamine in the hypothalamus also plays an inhibitory role in the release of prolactin and the negative feedback loop of this cycle as well where prolactin promotes secretion of dopamine [59]. Dopamine inhibits the pituitary release of prolactin, so the more dopamine present in the hypothalamus, the less prolactin will secrete and this prolactin negatively feeds back to increase dopamine secretion. Dopamine has a plethora of roles in many different systems and pathways, which can be detrimental to the body if dysregulation occurs due to specific environmental factors such as atrazine.

### 4.2. Serotonin

Serotonin is a neurotransmitter that has been associated with almost all types of behavior [60]. The neurons that synthesize this neurotransmitter are located in the midline of the brain stem, but project out to almost every other part of the brain [61]. The majority of the serotonin found in the body is found in the gastrointestinal tract, while a small amount, approximately 1–2%, is in the CNS [62]. Serotonin in the hypothalamus is linked to the regulation of food intake [63]. Studies have suggested that appetite is mediated through the arcuate nucleus and serotonin release within the hypothalamus suppresses appetite when needed [64].

Serotonin also has an active role in the activation of the HPA axis in the release of CRH, which further controls the release of ACTH [64]. Activated serotonin receptors in the brain cause an increase in CRH into the anterior pituitary resulting in an increase in the secretion of ACTH [65]. ACTH secretion then influences the release of glucocorticoids and androgens from the adrenal cortex. Cortisol is responsible for both the negative feedback loop to regulate CRH levels as well as assisting in the regulation of glucose metabolism and immune function [43]. The role of serotonin in the hypothalamus is important, because of its upstream position in many pathways, especially in this brain region.

### 4.3. Norepinephrine

Norepinephrine is a neurotransmitter that is primarily produced in the locus coeruleus of the brain stem and plays a role in the regulation of attention, arousal, and other cognitive behaviors. It is released from the neurons in this small brain region in response to stressful situations to many other brain regions through vast neuronal projections [66]. A stepwise synthesis occurs to produce the neurotransmitter starting with the amino acid tyrosine to synthesize dopamine. Dopamine-β-hydroxylase is then used to synthesize norepinephrine from dopamine [67]. The locus coeruleus and norepinephrine have a noticeable role in waking and arousal and when activated, even moderately, is not compatible with sleep state. Increased activity of the locus coeruleus is suggested to contribute to stress-related disorders [68]. The locus coeruleus is also known to have strong ties to the hypothalamus through the paraventricular nucleus, which is conceivably the main area of the brain that processes stressful stimuli experienced by the body. The axis between the locus coeruleus and the hypothalamus connects the autonomic nervous system with the CNS and the neuroendocrine system [69]. The links between the locus coeruleus, the hypothalamus, and the other systems listed suggest that dysregulation of norepinephrine or the systems in which it is involved can potentially have major adverse effects on the body. Norepinephrine is also a key regulator of the HPA axis and is an excitatory molecule that allows for secretion of CRH as well as ACTH dose-dependently [70,71]. Norepinephrine, in conjunction with dopamine, is also responsible for the inhibition of prolactin release and suppresses secretion dose-dependently [72]. Similar to dopamine, norepinephrine is involved in an array of different pathways, making it an important neurotransmitter to study in regard to environmental exposures that are known to affect the hypothalamus in particular.

### 4.4. Gamma-Aminobutyric Acid

Gamma-aminobutyric acid (GABA) is an inhibitory neurotransmitter in the mature brain, with neurons producing it in multiple areas of the brain, including the hypothalamus. GABA is synthesized from glutamate and function together as the antithesis of each other in the mature brain, where glutamate is the excitatory neurotransmitter [73]. GABA has an important role in the developing brain in processes involved in neurogenesis and also has the opposite function during this time period, being excitatory like glutamate rather than inhibitory [74]. In relationship to the hypothalamus’ function and pathways, GABA has been studied and shown to have relationship in regulation of ACTH, prolactin, gonadotropins, growth hormone, CRH, and a few others that are outside the scope of this review [75,76].

### 4.5. Glutamate

Glutamate, as mentioned previously, is the opposite of GABA in the mature brain and acts as an excitatory neurotransmitter. The body is very sensitive to the concentrations, location, and timing of secretion of this neurotransmitter to regulate excitatory signals, because of its role in multiple metabolic pathways [77]. The relationship between the hypothalamus and this excitatory neurotransmitter has been investigated and glutamate was discovered to be the primary excitatory agent that is released from the hypothalamic neurons. Since the hypothalamus is a regulator of the autonomic nervous system, neuroendocrine system, as well as multiple homeostatic functions, the role of glutamate in the hypothalamus is of extreme importance [78].

### 4.6. Acetylcholine

Acetylcholine is a very important neurotransmitter but in regard to the CNS, little is known. What has been discovered is that acetylcholine is released from the basal forebrain and the mesopontine tegmentum area and is mostly involved with memory, motivation, arousal, and attention [79]. Acetylcholine is distributed to the hypothalamus and has been investigated and thought to be the excitatory neurotransmitter of this area of the brain in absence of glutamate when the glutamate/GABA interactions are imbalanced [80]. This important finding suggests that acetylcholine takes over the responsibility that glutamate has in regulating all of the homeostatic functions of the hypothalamus when needed.

## 5. Effects of Atrazine on Major Neurotransmitters

As mentioned, atrazine is well known for being an endocrine-disrupting chemical, impacting the reproductive system due to alterations in release of hormones in that system. While this is an important area of study, there is also now research indicating that atrazine can impact neurotransmitters. These neurotransmitter alterations can also result in many downstream effects in the pathways in which they are involved, including some neuroendocrine pathways where neurotransmitter signaling is needed. Some of the key neurotransmitters have shown adverse effects in their concentrations or their signaling after different atrazine exposure scenarios in cellular models, rodents, and the zebrafish. Currently, most of the focus on atrazine’s impacts on neurotransmitters has been in other areas of the brain outside of the hypothalamus since generally the hypothalamus is known more for being a neuroendocrine structure. As such, neurotoxicity outcomes have been waived in recent human health risks assessments by the US EPA. Regardless, the effects that atrazine has on these neurotransmitters in other areas of the brain can play a role in the hypothalamic function and other pathways in which the hypothalamus is involved, warranting further research to define exposure limits for the neurotoxic outcomes.

### 5.1. Dopamine

Atrazine’s impacts on dopaminergic pathways in other brain regions beyond the hypothalamus are extensively studied. Although the hypothalamus is one of the main areas where dopamine is produced, research on dopamine dysfunction, including changes in concentration, is focused on the parts of the brain involved in the behaviors and processes listed previously. Therefore, there is a gap in the literature when it comes to atrazine exposure and the effects on the tuberoinfundibular pathway and dopamine that is produced and synthesized in the hypothalamus. However, there have been cell model studies conducted that can provide insight into the general effects on dopamine (and norepinephrine) in the brain that suggests that those outcomes are applicable to the hypothalamus specifically (Table 2). A study conducted with PC12 cells investigated the metabolism of dopamine after atrazine exposure and measured both intracellular dopamine concentrations as well as norepinephrine. It was discovered that exposure to atrazine, at concentrations ranging from 0 to 200 µM, altered the ability of the cells to synthesize these catecholamines with a decrease in both dopamine and norepinephrine intracellularly in a concentration dependent manner [81]. Atrazine’s adverse effect on dopamine in the hypothalamus specifically can also be supported by another study that concluded that the hypothalamus is a reasonable target of atrazine, because of the adverse effects that atrazine exposure had on secretion of prolactin, which is regulated by hypothalamic dopamine [82]. Decreased dopamine in the hypothalamus not only affects the release of prolactin, but also the negative feedback loop of this cycle as well where prolactin promotes secretion of dopamine [59]. It is important for hypothalamic dopamine to be studied more closely in regard to atrazine exposure, because of its importance in pathways associated with the neuroendocrine system.

As mentioned, there are many studies that look at areas of the brain other than the hypothalamus where dopamine is synthesized and those corresponding pathways after atrazine exposure. For example, multiple studies have been conducted on striatal dopamine [31,83]. There are also other brain regions that experience dopamine dysregulation that are more closely connected to the adverse outcomes of the hypothalamus that explain some of the behavior that is seen following atrazine exposure. For instance, dopamine levels in the hippocampus, which serves as a connection in the mesolimbic pathway to transport dopamine from the VTA to the nucleus accumbens, were examined [55]. In this study, rats were exposed to atrazine during developmental stages and impacts on hippocampal dopamine levels as well as mRNA and expression levels of D1 dopamine receptor were determined. Here, it was reported that in the low dose atrazine-exposed group of 10 mg/kg body weight (current acute dietary NOAEL), dopamine levels were increased in the hippocampus compared to the control and the high dose group of 100 mg/kg body weight, while D1 dopamine receptor expression and mRNA levels responded in a decreasing dose-dependent manner [53]. The increase in dopamine at the low dose was attributed to the compensatory action of the dopaminergic neurons to the atrazine exposure, because the decrease in dopamine in the higher concentration exposure was comparable to a previous study [31]. Further studies confirming lower concentrations of dopamine after exposure to atrazine up to 100 mg/kg have also looked at the association of reduced gene expression of Nurr1 and VMAT2 in pubertal rats as well as juvenile rats exposed in utero, which are both associated with dopaminergic pathways [84,85,86]. In addition, it was observed that a developmental atrazine exposure at 10 or 100 mg/kg in male Sprague Dawley rats led to hippocampal lesions and suggests that MEK/ERK/CREB cycles may play a role in the effects seen [87]. Furthermore, striatal dopaminergic pathways were also identified to be responsible for inducing apoptosis and autophagy amongst dopaminergic neurons, potentially causing these changes in dopamine concentration that were reported in the previously mentioned studies [88]. Rat striatal slices exposed to atrazine from adult male Sprague Dawley rats provided evidence that the decreases in dopamine are most likely due to atrazine affecting the storage or cellular uptake of the neurotransmitter [89,90]. This hypothesis was further confirmed by a study looking at the striatal vesicles, which saw an increase in cytosolic dopamine, suggesting issues regarding neurodegenerative pathologies [91]. Cell models are also providing evidence that atrazine exposure between 1 and 250 µM can cause phenotypic changes that are associated with Parkinson’s Disease. As such, it is important to continue to study the effects that atrazine has on the brain and the CNS, because other neurodegenerative diseases may also be of concern [91].

Numerous studies have been conducted completely independent of the hypothalamus that show dysfunction in the dopaminergic pathway of the striatum leading to a variety of adverse effects. Chronic exposure to atrazine at 10 mg/kg is reported to affect locomotor activity, impair motor coordination, and impair learning tasks, but did not affect concentration of dopamine in the hypothalamus in the male Sprague Dawley rat [92]. These outcomes are supported by another study in the same rodent model that found that repeated atrazine exposure at 100 mg/kg caused a decrease in dopamine as well as locomotor activity [93]. This study also reported that after function returned to normal following exposure, an amphetamine challenge caused drastic stimulation of the dopaminergic system, suggesting the reasoning for certain behavior outcomes observed after atrazine exposure [93]. Non-dopaminergic neurons were also studied and were associated with a decrease in locomotor activity at a dose of 100 mg/kg, supporting the fact that atrazine causes a general hypoactive effect even after acute exposures [94]. Furthermore, subtoxic, environmentally relevant doses of atrazine, at 1 and 100 µg/kg (0.001 and 0.1 mg/kg), were administered perinatally to CD1 mice and resulted in subtle functional and behavioral changes [95]. These behavior outcomes seen at different time points in development provide insight into the many effects that atrazine can have based on timing of exposure.

**Table 2:** Reported adverse effects on dopaminergic systems following atrazine exposure.

Reference	Species	Atrazine Exposure ^a^	Length of Exposure	Results
Das et al., 2000 [81]	PC12 Cells	12.5, 25, 50, 100, or 200 µM	6, 12, 8, 24, 48 h	Decrease in intracellular DA, concentration dependent outcomes, NE reduction at 100 and 200 µM
Coban and Filipov, 2007 [31]	Male Juvenile C57BL/6 Mice	5, 25, 125, or 250 mg/kg	14 days, gavage	Dose-dependent decrease of DA in striatum up to one week after exposure
Walters et al., 2015 [83]	Male Sprague-Dawley Rats	0.1 or 10 mg/kg	Dams treated Gestational Day 1- Postnatal Day 21, Male offspring continued 6 months upon weening	Male offspring showed decrease in DA and DOPAC levels at low and high concentrations, 10 mg/kg showed disruptions in motor function
Li et al., 2018 [53]	Sprague Dawley Rats	10 or 100 mg/kg	30 consecutive days starting at Postnatal Day 28, gavage	DA levels increased in hippocampus in low dose group, D1DR expression levels decreased in dose-dependent manner
Li et al., 2015 [84]	Pubertal Male Sprague Dawley Rats	50, 100, or 200 mg/kg	28 days, gavage, postnatal day 27–54	Exposure to higher doses led to decreased levels of DA and decreased expression of Nurr1
Sun et al., 2014 [85]	Sprague Dawley Rats	Pregnant Dams received 10 µL/g body weight or vehicle, offspring received 0, 25, or 50 mg/kg/day	Pregnant dams starting GD 5, offspring until PND 22, gavage	Ventral midbrains examined and found DA concentrations and mRNA of Nurr1 decreases in offspring at both concentrations
Li et al., 2014 [86]	Sprague Dawley Rats	25 or 50 mg/kg/day	Pregnant dams received ATR from GD 0, and offspring received ATR until PND 1	6 months after treatment DA and expression of Nurr1 were decreased in striatum and substantia nigra
Li et al., 2019 [87]	Male Sprague Dawley Rats	10 or 100 mg/kg	30 days, starting at PND 35	Impairment of memory after exposure, downregulation of protein and mRNA expression levels associated with MEK/ERK/CREB pathway at low and high concentrations
Song et al., 2015 [88]	Male Wistar Rats	10, 50, or 100 mg/kg	3 months, gavage	Nigrostriatal dopaminergic system pathways examined and found micromorphology suggesting neuronal apoptosis and mitochondrial autophagy of DA neurons at all concentrations, increasing in severity with increasing dose
Filipov et al., 2007 [89]	Adult Male Sprague Dawley Rats	up to 500 µM	Striatal slices incubated for 4 h	Tissue DA levels decreased dose-dependent at 100 µM or greater, exposure interferes with the uptake and storage of DA
Hossain and Filipov, 2008 [90]	Striatal synaptosomes and vesicles from Adult Male Sprague Dawley Rats	1–250 µM	15 min	Atrazine and two metabolites caused inhibition of DA uptake dose-dependently
Bardullas et al., 2011 [92]	Male Sprague Dawley Rat	10 mg/kg	1 year	Impaired motor coordination, greater spontaneous locomotor activity, decrease in striatal DA
Rodríguez et al., 2013 [93]	Adult Male Sprague Dawley Rat	100 mg/kg	6 IP injections over 2 weeks	Hypoactivity following injection that lasted for 5 days, reductions in striatal DA, amphetamine 2 months post exposure caused significant stimulation
Rodriguez et al., 2017 [94]	Male Sprague Dawley Rats	100 mg/kg	Single injection	Significant decrease in locomotor activity, cells other than DA neurons cause locomotor dysfunction
Belloni et al., 2011 [95]	Juvenile and Adult CD1 Mice	0.001 or 0.1 mg/kg	Dams exposed GD 14 through PND 21	Feminization of behavioral profile in male offspring, learning performance alterations in adults at both concentrations

^a^ NOAEL is 10 mg/kg/day and LOAEL is 70 mg/kg/day for acute dietary exposure in females aged 13–50 years. Residential NOAEL is 6.25 mg/kg/day and LOAEL is 12.5 mg/kg/day, which accounts for infants and children. For chronic dietary exposure for all ages, NOAEL is 1.8 mg/kg/day and LOAEL is 3.65 mg/kg/day based on 6-month rat study.

### 5.2. Serotonin

Impacts on serotonin following atrazine exposure have not been extensively investigated, but some studies report dysregulation. Alterations in the serotonergic system and associated pathway genes were reported in adult female zebrafish following an embryonic atrazine exposure at 0.3, 3, or 30 ppb (µg/L) [96]. Further, in juvenile and adult offspring of C57BL/6 mice exposed at an estimated 1.4 mg/kg/day, which is below the current chronic dietary NOAEL, showed a significant decrease in serotonin of the perirhinal cortex in only female adult offspring and was linked to a deficiency in memory. This conclusion was confirmed in the novel object recognition test [97]. In zebrafish, it was also observed that paternal atrazine exposure at 0.3, 3, or 30 ppb had intergenerational dysfunction of the serotonergic system in the offspring [98]. Although only a few limited studies have been completed, each gives support that atrazine may interfere with the serotonergic system with potential for sex-specific responses at doses near or below the current chronic dietary NOAEL at 1.8 mg/kg/day.

### 5.3. Gaba and Glutamate

Similar to serotonin, there have not been many studies on the effects of atrazine exposure on GABA or glutamate. Typically, these two neurotransmitters are studied together, because they act in concert in the brain and one is a precursor to the other. One study in male albino rats did conclude that there were increases in glutamate and GABA in several areas of the brain, but not specifically in the hypothalamus after atrazine exposure at 1 or 10 mg/kg [99]. This study suggests that there may be alterations of these concentrations in other areas of the brain as well, such as the hypothalamus, and at different points during development where GABA plays a different role. Conversely, in zebrafish no significant changes in GABA were observed in the brain of adult males or females that were exposed to 0.3, 3, or 30 ppb atrazine during embryogenesis [96]. It is difficult to conclude at this time the relationship between atrazine exposure and GABA/glutamate given the limited studies, but similar to other neurotransmitter systems further investigation is needed to determine potential impacts in the different brain regions.

### 5.4. Acetylcholine

Acetylcholine is also a neurotransmitter that has not been extensively studied with atrazine exposure. There has been one study that utilized the zebrafish model to examine acetylcholinesterase activity in the brain following atrazine exposure. Here, both acetylcholinesterase (AchE) activity in muscles and in the brain were investigated, but alterations were only detected in the brain. The reduction in AchE in the brain and the behavior outcomes that were also observed in this study after atrazine exposures of 10 or 1000 µg/L provide proof that atrazine may affect defensive behaviors in the zebrafish, which can be associated with an issue in neurotransmission of acetylcholine, described by the reduction in the AchE activity [100]. Overall, more studies are needed to further understand the influence of atrazine on this neurotransmitter.

## 6. Relevance to Human Exposure

A clear indication of human atrazine exposure is complicated by limitations in accurate drinking water monitoring data and confines in biomonitoring assessments. These drawbacks present difficulty when working to translate doses and concentrations used in laboratory studies of relevance to human health. According to the United States Centers for Disease Control and Prevention (US CDC), it is unknown whether the atrazine concentrations that are detected in the environment, and the levels that are observed via biomonitoring, result in adverse human health effects. The US CDC also acknowledges that particular attention needs to be paid to seasonal concentrations of atrazine in the drinking water sources of communities in regions where atrazine is applied on agricultural fields, where these atrazine concentrations can be higher than the limits set by the US EPA (i.e., greater than 3 ppb in drinking water) [101]. This is an important factor when discussing exposure assessments since atrazine concentrations can vary depending on time of year and this is not always noted in these studies. It should also be recognized that individuals residing in rural communities in regions where atrazine is applied are likely to rely on private drinking water wells, which are not routinely monitored for chemical contaminants. Furthermore, while the US CDC claims that the general population is not as frequently exposed to atrazine through their drinking water, a study evaluating urinary profiles to test for atrazine metabolites demonstrated drastically different metabolite profiles, suggesting that multiple metabolites need to be assessed to reflect atrazine exposure, rather than single evaluation of the parent compound and/or of one main metabolite [101,102]. Barr et al. reported that the percent distribution of atrazine metabolites in human urine varies with the extent of exposure (i.e., high or low acute exposures or environmental exposures) [103]. Specifically, the environmental exposures resulted in a distribution of 77% diaminochlorotriazine (DACT), 15% desethylatrazine (DEA), 6% deisopropylatrazine (DIA), and 2% atrazine mercapturate [103]. This is another important point to consider as the US CDC biomonitoring information available suggests that recent exposure assessments are only measuring the urinary levels of atrazine mercapturate [101]. Atrazine mercapturate was detected at the lowest percent compared to the other major metabolites and thus, is very likely not accurately representing atrazine body burden and exposures in the US population.

In the environment, atrazine’s half-life is different in both soil and water systems and can vary depending on pH, exposure to sunlight, oxygen level, as well as climate conditions. In soil, the half-life of atrazine is between 60 and 75 days, while in water with direct sunlight it is 168 days. Atrazine’s great potential for reaching both ground and surface water is a major cause for concern and has prompted epidemiological studies to investigate if there are differences among populations in communities where atrazine use is more prevalent as opposed to areas with less atrazine use [103]. Overall, to understand atrazine exposure risks in humans, it is important to consider atrazine’s chemical properties in a variety of environmental conditions, which will reflect likelihood of atrazine contaminating drinking water sources and thus, lead to environmental human exposures. In addition, consideration of the time of year of exposure assessments informs on higher risks of exposure to atrazine at elevated concentrations in drinking water sources, since concentrations are known to spike in drinking water sources following the first major rain event after agricultural field application. Unfortunately, it was discovered that many community drinking water systems in the Midwestern US aware of this seasonal fluctuation were strategically testing atrazine during certain months to avoid identification of the chemical spike to alleviate economic burden associated with removing atrazine from the drinking water. This atrazine water testing strategy met the previous US EPA drinking water testing requirements, but the spikes in atrazine concentrations in drinking water sources and subsequently the risk of human exposure to higher atrazine concentrations were still occurring. This concern was recognized when the former owner of atrazine, Syngenta (ChemChina purchased Syngenta in 2017), was required to fund additional atrazine water monitoring during higher risk months for a more informed record of atrazine concentrations in drinking water during these time periods. This increased water monitoring also gave a more accurate understanding on when and if actions were needed to remove atrazine from community drinking water sources, when the chemical was detected at concentrations above the US EPA MCL and ultimately decrease risk of human exposure. In contrast, in 2020 the US EPA relaxed these regulations allowing for 50% more atrazine to enter water bodies and suspended the requirement for this more frequent water testing. As such, increased atrazine concentrations in drinking water sources are again likely and present higher risks of environmental atrazine exposure. In summation, it can be concluded that multiple limitations are present in our current knowledge for accurate estimates of risks of environmental atrazine exposure in the general population, which is further complicated by current exposure assessments measuring only atrazine mercapturate not representing the full atrazine body burden and exposures in humans.

Regardless of these limitations, it is important to work to translate doses/concentrations at which observed adverse health outcomes are observed in laboratory studies. Given the challenges detailed in the previous paragraphs, there are questions regarding whether the US EPA benchmark dose limit of 2.42 mg/kg per day for four days poses a risk to human health. This value was anchored to atrazine’s effect on LH as the US EPA recognizes atrazine’s impacts on GnRH regulation of LH release as the most sensitive endpoint for risk assessment [103]. The health outcomes discussed above from a plethora of atrazine studies provide evidence that there are likely many integrated pathways altered by atrazine exposure, resulting in the multiple different health outcomes identified in these studies including neurotoxicity, which has been waived from inclusion in the most recent atrazine human health risk assessments [103]. As such, the argument can be made that impacts on LH should not necessarily be the sole indicator of toxicity in regard to atrazine regulation.

There is also evidence that atrazine is persistent in soil and water dependent on environmental conditions. In countries where atrazine use has been banned for quite some time, this chemical is still found to be the most abundant pesticide in both soil and groundwater, presenting continual risks for exposure [104]. Similarly, environmental monitoring in countries where atrazine is still applied continues to detect atrazine at concentrations above the US EPA regulatory limit of 3 ppb in drinking water sources [4]. Atrazine removal strategies may not be implemented and/or may miss chemical spikes based on time of water testing as noted above. On the global scale, it should also be remembered that the World Health Organization regulatory limit was revised and increased to 100 ppb, again allowing for higher atrazine exposure concentrations in drinking water. These combined characteristics of atrazine in the environment; testing, detection, and removal strategies; and variations in drinking water regulatory limits globally are critical when assessing the potential for atrazine exposure and health risks in humans.

When considering the atrazine doses/concentrations of many of the mammalian studies discussed above, we see that many do have very similar ranges. Although the concentrations used to dose in the in vivo studies do not align with the exact concentrations that are found in the drinking water or soil, these concentrations must be converted into tissue dose received in the organism. Unfortunately, a common limitation of these studies is that they do not include the tissue dose or urinary concentrations of atrazine or atrazine metabolites after exposure. Some of the studies, though, include exposures with the individual atrazine metabolites including those focused on the HPA axis. Overall, these studies conclude that the major atrazine metabolites have similar toxicity or are less toxic than the parent chemical [27,28,49]. It does appear that mammals and fish quickly metabolize atrazine into the major metabolites, suggesting that it may be a combination of the parent compound and metabolites that cause many of the adverse health outcomes observed after atrazine exposure. It is also known that the major metabolites of atrazine are still detected in the urine of mice 48 h after administration of the dose [105]. These toxicokinetic characteristics are important to recognize when comparing the concentrations found in the environment and the doses used in controlled studies for a more informed approach in translating these laboratory studies.

In addition to centering atrazine exposure concentrations to the drinking water regulatory level in toxicity tests, atrazine doses used across the different model systems are many times determined by the no observed adverse effect level (NOAEL) and/or the lowest observed adverse effect level (LOAEL). As noted above, the US EPA NOAEL for atrazine is 10 mg/kg/day, while the LOAEL is 70 mg/kg/day for acute dietary exposure (females aged 13 to 50 years) based on delayed ossification of cranial bones in fetuses observed in developmental toxicity studies in rat and rabbit [106]. These oral short-term dietary values are slightly adjusted for residential human health risk, which considers protection of infants and children (i.e., NOAEL is 6.25 mg/kg/day and LOAEL is 12.5 mg/kg/day). For chronic dietary exposure, the NOAEL is 1.8 mg/kg/day and the LOAEL is 3.65 mg/kg/day based on attenuation of the pre-ovulatory LH surge reported in a 6-month study in rats for all [106]. Nonetheless, as mentioned above, some studies have observed neurotoxic outcomes at doses that are lower than the NOAELs reported by the US EPA. As such, further studies are warranted that focus on neurotoxicity outcomes to determine if significant impacts may be observed at doses lower than those based around atrazine’s impact on LH.

Atrazine is not thought to bioaccumulate, but it is not yet known where in the body atrazine is distributed following absorption. From the above discussed studies, we do know that atrazine exposure impacts the brain, including the neuroendocrine system and the regions of focus in the dopaminergic studies. The overall research body to date supports a yet to be identified neuroendocrine target of atrazine toxicity (specifically hypothalamic target) with neuroendocrine dysfunction and neurotoxicity outcomes likely the most susceptible systems, but other impacts such as immunotoxicity have been reported. It is not yet well understood whether other systems or specific tissues are more susceptible. If these limitations were addressed in future research, this would provide a more holistic understanding of the basics of atrazine toxicity beyond the many adverse health outcomes that are reported. We do know that there is substantial crosstalk among the endocrine and CNS. Recognition of these integrated pathways provides support of how atrazine could impact the multiple endocrine axes and CNS outcomes and suggests mechanisms by which this may occur.

## 7. Crosstalk between CNS and Endocrine System

Crosstalk amongst different systems has been speculated between multiple physiological systems and may provide evidence as to why there are numerous adverse health outcomes reported following atrazine exposure when the hypothalamus seems to be the main target. The HPA and HPG axes work in concert to reduce allostatic load and if disrupted, can have effects later in life such as anxiety, depression, and post-traumatic stress disorder [29]. The outcomes discussed with regard to atrazine exposure and these two hypothalamic axes provide reason to speculate that the crosstalk between these two pathways is occurring and influencing each pathway when atrazine reaches the brain.

**Figure 3:**
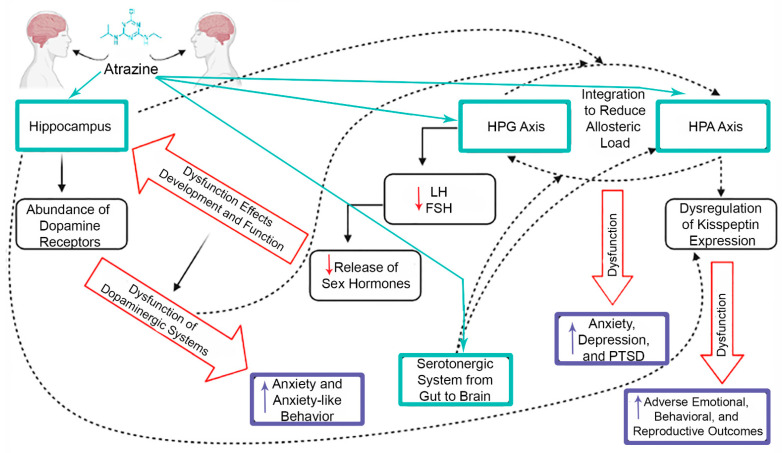
Targets of atrazine in the web of intertwined pathways that communicate through crosstalk among the endocrine axes and the central nervous system. Proximity of the hypothalamus and the hippocampus allow for communication between the different systems. Both systems are involved in explaining the adverse behavioral, reproductive, neurological, and endocrine outcomes associated with atrazine exposure. Dotted arrows represent the communication across systems and with which components of these pathways cause the various dysfunctions. Teal boxes and arrows represent the targets of atrazine, while the black boxes illustrate the dysfunction that occurs in these systems and pathways after exposure to atrazine (decreases in LH, FSH, and release of sex hormones are indicated with red arrows). The purple boxes represent the outcomes that are observed resulting from the dysfunction that occurs within the systems (increases in these outcomes are indicated with purple arrows). Image created in BioRender.

The hippocampus is not extensively studied in regard to atrazine exposure, but there are a few studies that have discussed observed effects, which can help elucidate the wide array of different outcomes, from behavior to reproduction, following atrazine exposure. The position where the hippocampus resides in the brain allows it to be easily influenced by the dysfunction that the surrounding brain regions experience following atrazine exposure as well. Many studies confirm that sex hormones play a role in the developing brain outside of the hypothalamus, which is typically associated with reproductive function. Sex hormones that are regulated by FSH and LH are speculated to affect the development and function of the hippocampus, which can lead to impairments in cognitive function [107]. This coincides with the previously mentioned atrazine study on the hippocampus citing impairments to spatial memory and hippocampal D1 dopamine receptor [53]. LH and FSH regulate the production and release of both estrogen and progesterone, which have an abundance of receptors in the hippocampus and surrounding brain regions [44,107]. The hippocampus’ location allows it to connect with other regions of the brain, including the hypothalamus and striatum, which are two main regions involved in the dopaminergic systems [55,56]. The hippocampus can therefore easily interact with regions close by, but more importantly can also be affected by the dysfunction that these regions experience. Dopamine receptors in the hippocampus are in abundance and play a role in anxiety behavior [108]. This relationship was observed and confirmed in regard to atrazine exposure through behavioral endpoints analyzed in rats where anxiety-like behavior was linked to a dysregulation in dopaminergic systems [32]. Although each part of the brain is involved in distinct processes and have specific functions, the proximity to other areas of the brain and the interweaving of different systems through the brain is why crosstalk is a large factor to consider when trying to find a central target for atrazine toxicity. This is especially true when considering neurotransmitters and the hypothalamus, because many of the neurotransmitters have high priority functions and areas of the brain that they are associated with, but the interactions with the hypothalamus are still relevant, especially when looking at the multitude of adverse health outcomes observed after atrazine exposure across many organs and systems (Figure 3).

One recently identified upstream target of atrazine is the kisspeptin system. Kisspeptin is a family of hormones that are generated from the KISS1 gene that is emerging as a potential target of atrazine due to its role in the HPG axis as well as in other parts of the brain. This target may provide explanation as to why atrazine causes a wide array of outcomes, from reproductive to emotional and behavioral, across different systems [109]. Kisspeptin has been proven to alter prolactin secretion, which then affects the dopaminergic system stemming from the hypothalamus [110,111]. The adverse outcomes in the hippocampus after exposure to atrazine that can cause anxiety-like behavior might also be associated with kisspeptin as a target. The literature suggests that kisspeptin expression is intertwined with the HPA axis and its response to stressors [112]. Crosstalk that occurs between close brain regions such as the HPA axis of the hypothalamus and the hippocampus, mentioned previously, has the potential to induce anxiety-like behavior in the hippocampus through kisspeptin [45,46]. These studies demonstrate how dysfunction in different neurons in the brain can be linked to the dysfunction in the neuroendocrine system, also leading to certain behaviors. This is the reason for speculation that the hypothalamus may be the central target of atrazine, because many of the adverse outcomes seen can be traced back to that area of the brain even if it is indirectly.

Serotonin in general has not been a focus of concern in regard to atrazine exposure, but some of the studies that are available provide evidence that gut serotonin is affected by lower concentrations of atrazine in the male [113]. This type of dysfunction has the potential to have an impact on the brain, specifically the arcuate nucleus in the hypothalamus, or stem from dysfunction in the hypothalamus due to the evidence of crosstalk between the gastrointestinal system and this area of the brain [114]. The serotonergic system and the HPA axis also experience crosstalk based on location in the brain and dysfunction that may occur in either or both of these systems and can lead to anxiety-like disorders [115]. These studies and the crosstalk that seems to occur between these systems is particularly interesting because not only are the endocrine and nervous systems showing behavioral outcomes that result from imbalances in the brain, but that other systems in the body that are not proximal to the brain can also be affected and linked to the same region of the brain that is of concern regarding atrazine exposure. All of the studies discussed in this review allow for multiple hypothetical mechanisms in which atrazine toxicity occurs that seem to be intertwined based on the evidence of crosstalk between the systems and pathways that atrazine exposure is likely to affect.

## 8. Conclusions

Atrazine usage in the United States and other global regions is of public health importance not only because of the implications of atrazine as an endocrine-disrupting chemical and reproductive toxicant, but also as a neurotoxicant and impacts on neurotransmission. The augmentation in hormone secretion necessary for reproduction and endocrine function that is regulated by the hypothalamic axes is prevalent in multiple animal models and changes seen after atrazine exposure are observed in both sexes. There is reason for concern across multiple organisms, multiple routes of exposure, multiple stages of development when exposure occurs, and the potential of multigenerational effects. There is a wide array of adverse health outcomes that have been discovered and studied regarding atrazine exposure in many animal models, suggesting that there must be crosstalk within systems in the brain and with the reproductive system, likely stemming from one single target. Other neurotransmitters that are outside the scope of this review may also have the potential to be affected by atrazine exposure and their relationship with the current known systems that are altered by atrazine. The effects seen based on the current research and the complex systems that weave together a mechanistic web of dysfunction in the body provide evidence to consider atrazine as not only of ecological concern, but of public health concern as well. Furthermore, the current regulatory levels for human health are centered on the NOAELs of atrazine’s attenuation of the LH surge. The US EPA identifies this mechanism as the most sensitive and the most recent human health risk assessments have not included neurotoxicity outcomes as these are generally investigated separate from the neuroendocrine parameters. Recent neurotoxicity studies have identified alterations at doses near and/or below these NOAELs, warranting further evaluation and consideration of the intertwined neuroendocrine and central nervous systems.

## Figures and Tables

**Table 1:** Atrazine adverse impacts reported along the HPG axis.

Reference	Species	Atrazine Exposure ^a^	Length of Exposure	Results
Foradori et al. [8]	Ovariectomized Adult Female Wistar Rats	200 mg/kg	4 days, gavage	Reduction in GnRH pulse frequency at 200 mg/kg
Foradori et al. [9]	Ovariectomized Adult Female Wistar Rats	50, 100, or 200 mg/kg	4 days, gavage	Reduction in LH and FSH surges at all concentrations and at 200 mg/kg, respectively due to decrease in GnRH neurons in 100 mg/kg and 200 mg/kg concentrations, 4 days after treatment GnRH neuronal function returned to normal
Goldman et al. [45]	Ovariectomized, Estrogen-Primed Long Evans Hooded Female Rats	10, 30, or 100 mg/kg	4 days or single administration	Suppressed LH surge after four daily treatments at 100 mg/kg, elevations in LH surge after single administration of 10, 30, and 100 mg/kg
Kimura et al. [46]	Ovariectomized Female Wistar Rats	100 mg/kg	5 days, orally	Reduced LH surge and *Kiss1* mRNA expression in AVPV
Trentacoste et al. [47]	Peripubertal Male Sprague Dawley Rats	1–200 mg/kg	Day 22–47, gavage	100 and 200 mg/kg reduced serum and intratesticular testosterone, reduced serum LH
Wirbisky et al. [26]	Zebrafish	0.3, 3, or 30 ppb (µg/L)	1–72 hpf	Increase in progesterone at 3 and 30 ppb, increase in follicular atresia
Mokhtari et al. [48]	Male Wistar Rats	100, 200, or 400 mg/kg	14 days intraperitoneally	Serum LH decreased in 200 and 400 mg/kg group, FSH decreased in 400 mg/kg group, decrease in testosterone in experimental groups

^a^ NOAEL is 10 mg/kg/day and LOAEL is 70 mg/kg/day for acute dietary exposure in females aged 13–50 years.
